# Influence of Radiotherapy Timing on Sealer-Dentin Adhesion: A Confocal Microscopy and Push-Out Bond Strength Study

**DOI:** 10.3290/j.jad.c_2792

**Published:** 2026-08-31

**Authors:** Gamze Nalci Calik, Burcu Oglakci Ozkoc, Ceren Deger, Humeyra Capkin, Kerime Akdur, Alpaslan Mayadagli

**Affiliations:** a Gamze Nalci Calik Assistant Professor, Bezmialem Vakif University, Faculty of Dentistry, Department of Endodontics, Istanbul, Turkiye. Idea, experimental design, performed the experiments, wrote the manuscript.; b Burcu Oglakci Ozkoc Associate Professor, Bezmialem Vakif University, Faculty of Dentistry, Department of Restorative Dentistry, Istanbul, Turkiye. Hypothesis, experimental design, proofread the manuscript, wrote the manuscript.; c Ceren Deger Assistant Professor, Bezmialem Vakif University, Faculty of Dentistry, Department of Restorative Dentistry, Istanbul, Turkiye. Performed the experiments, wrote the manuscript, proofread the manuscript.; d Humeyra Capkin Research Assistant, Bezmialem Vakif University, Faculty of Dentistry, Department of Endodontics, Istanbul, Turkiye. Performed the experiments, wrote the manuscript.; e Kerime Akdur Medical Radiophysic Specialist, Bezmialem Vakıf University, Faculty of Medicine, Radiation Oncology Department, Istanbul, Turkiye. Performed the irradiation procedures.; f Alpaslan Mayadagli Professor, Bezmialem Vakıf University, Faculty of Medicine, Radiation Oncology Department, Istanbul, Turkiye. Responsible for the radiotherapy protocol and contributed to the methodological design and discussion of the results.

**Keywords:** bioceramic sealer, bond strength, confocal laser scanning microscopy, dentin, push-out, radiotherapy, tubule penetration

## Abstract

**Purpose:**

The purpose of this study was to evaluate the influence of radiotherapy timing on the dentinal tubule penetration and push-out bond strength of an epoxy resin-based sealer (AH Plus) and a bioceramic sealer (BioRoot RCS).

**Methods and Materials:**

In total, 180 single-rooted sound human mandibular premolars were used and allocated to three main groups with different radiotherapy timings: Control (no irradiation), Before RT (irradiation prior to root canal obturation), and After RT (irradiation after root canal obturation). Subsequently, each group was subdivided into different sealer types: AH Plus and BioRoot RCS. For the Before RT and After RT groups, a cumulative dose of 60 Gy (6 weeks, 2 Gy per day, 5 days per week) was delivered. The dentinal tubule penetration depth was assessed using confocal laser scanning microscopy (n = 15), and the push-out bond strength was measured using a universal testing machine (n = 15). The data were analyzed using three-way ANOVA and Bonferroni tests (*p* < 0.05).

**Results:**

In the coronal thirds, the After RT group showed significantly lower tubule penetration and bond strength compared with the Control group for both sealers. In the apical thirds, BioRoot RCS was predominantly affected. The After RT group also showed significantly lower bond strength and tubule penetration than the Before RT group. These results were confirmed by the higher frequency of adhesive failures in the After RT group compared to the others. Finally, in the coronal thirds, AH Plus exhibited significantly higher bond strength than BioRoot RCS despite having lower dentinal tubule penetration.

**Conclusion:**

Radiotherapy timing significantly influenced sealer-dentin adhesion, varying by root canal levels and material types. Radiotherapy applied after obturation adversely affected the sealer-dentin interaction, resulting in reduced tubule penetration and bond strength, especially for BioRoot RCS.

Each year, approximately 2.4 million new cases of head and neck cancer (HNC) are diagnosed worldwide, resulting in nearly 1.3 million deaths.^33^ The management of HNC commonly involves surgery in combination with radiotherapy, either as a standalone treatment or as part of multimodal protocols that may also include chemotherapy.^16^ Radiotherapy, which utilizes ionizing radiation as the therapeutic modality, plays a central role in improving disease control and survival outcomes in these patients.^7^


The increasing survival rates of patients with HNC have, consequently, drawn greater attention to the development of evidence-based dental care protocols, particularly in relation to the use of radiotherapy.^28^ Indeed, as dental practitioners more frequently encounter patients undergoing or who have completed radiotherapy, understanding its potential impact on endodontic procedures has become increasingly important. Ionizing radiation has been reported to induce structural and chemical alterations in dental hard tissues, especially dentin, which contains a substantial amount of water.^14,17,23,35
^ Radiation-induced damage to these hard tissues is primarily attributed to radiolysis and the formation of free radicals, which lead to the degradation of the collagen matrix, dentin dehydration, alterations in dentin composition, and possible mineral precipitation within the dentinal tubules, resulting in tubule occlusion.^17,23,35
^ These changes may adversely affect the mechanical properties of dentin, such as microhardness and adhesion, thus making irradiated teeth more susceptible to pulpal and periapical pathologies.^32,37
^


Successful root canal treatment depends on the thorough cleaning and shaping of the root canal system, followed by proper 3D obturation. From a biological perspective, the quality of the obturation is closely related to the ability of the sealing materials to penetrate the dentinal tubules, as this penetration increases the contact surface area between dentin and the filling material and improves the sealing ability of the root canal system.^12,20,36
^ In addition, the adhesion of root canal sealers to the dentinal walls is critical for maintaining the integrity of the sealer-dentin interface and preventing microleakages and reinfection. As such, the push-out bond strength is considered a clinically relevant parameter, as it reflects the resistance of the root canal filling materials to dislodgement from dentin.^11^ Overall, both the dentinal tubule penetration and the push-out bond strength are important indicators of the sealing performance of root canal sealers and their potential for long-term endodontic success.^12,20
^


Among the currently available sealers, epoxy-resin-based materials such as AH Plus (Dentsply Sirona, York, PA, USA) have been widely used due to their good adhesion to dentin and penetration into the dentinal tubules, which are largely attributed to chemical interactions with the collagen matrix.^34^ More recently, bioceramic sealers containing calcium silicate such as BioRoot RCS (Septodont, Saint-Maur-des-Fossés, France) have been introduced; these sealers are characterized by their hydrophilic nature, bioactivity, and ability to interact with dentin through both organic and inorganic components.^13^


Bodrumlu et al^2^ reported that the apical sealing ability of resin-based root canal sealers decreased slightly when radiotherapy was administered. Furthermore, Pandolfo et al^27^ recently demonstrated that the radiation dose and obturation technique significantly influenced the adhesive performance and sealer penetration of an epoxy-resin-based sealer. However, there is limited information available regarding how the timing of radiotherapy influences the dentinal tubule penetration and bonding performance of different root canal sealers. Therefore, the aim of this i*n vitro* study was to evaluate the influence of the timing of radiotherapy (ie, performed either before or after root canal obturation) on the dentinal tubule penetration and push-out bond strength of both an epoxy-resin-based sealer (AH Plus) and a bioceramic sealer (BioRoot RCS).

The null hypothesis was as follows:

The timing of radiotherapy would not affect the dentinal tubule penetration or push-out bond strength of the different sealers.

## Methods and Materials

### Sample Size Determination

The required sample size was calculated using G*Power software (Heinrich Heine University, Düsseldorf, Germany) for a three-way ANOVA design (fixed effects, special, main effects and interactions). Based on a large effect size (f = 0.40), a significance level of α = 0.05, and a statistical power of 90%, the minimum required sample size was determined to be 90 specimens (15 specimens per subgroup). This calculation was based on the primary outcome measures of the study, namely the push-out bond strength and confocal laser scanning microscopy (CLSM) evaluations.

### Specimen Selection

The study received approval from the Scientific Research Ethics Committee of Bezmialem Vakif University. A total of 180 recently extracted, sound, and anonymized human single-rooted mandibular premolars were selected for inclusion in the study. Teeth with open apices, multiple canals, moderate-to-severe curvature, pulp chamber calcifications, internal resorption, or previous endodontic treatment were excluded. The premolars were cleaned and stored in distilled water at 37°C to prevent dehydration until testing.

Figure 1 shows the experimental study design of this study. Table 1 shows the chemical compositions of the dental materials used and application protocols. A total of 90 specimens were used for the push-out bond strength testing (n = 15 per subgroup), and 90 were used for the CLSM analysis (n = 15 per subgroup). All of these specimens were randomly divided into six groups. Firstly, the specimens were randomly assigned to three main groups: 1) Control (no irradiation); 2) Before RT (irradiation administered prior to root canal obturation); and 3) After RT (irradiation applied after root canal obturation). Subsequently, each group was subdivided into two groups with different sealer types: AH Plus (epoxy resin-based, Dentsply Sirona, York, PA, USA) or BioRoot RCS (tricalcium silicate-based, Septodont, Saint-Maur-des-Fossés, France).

**Fig 1 Fig1:**
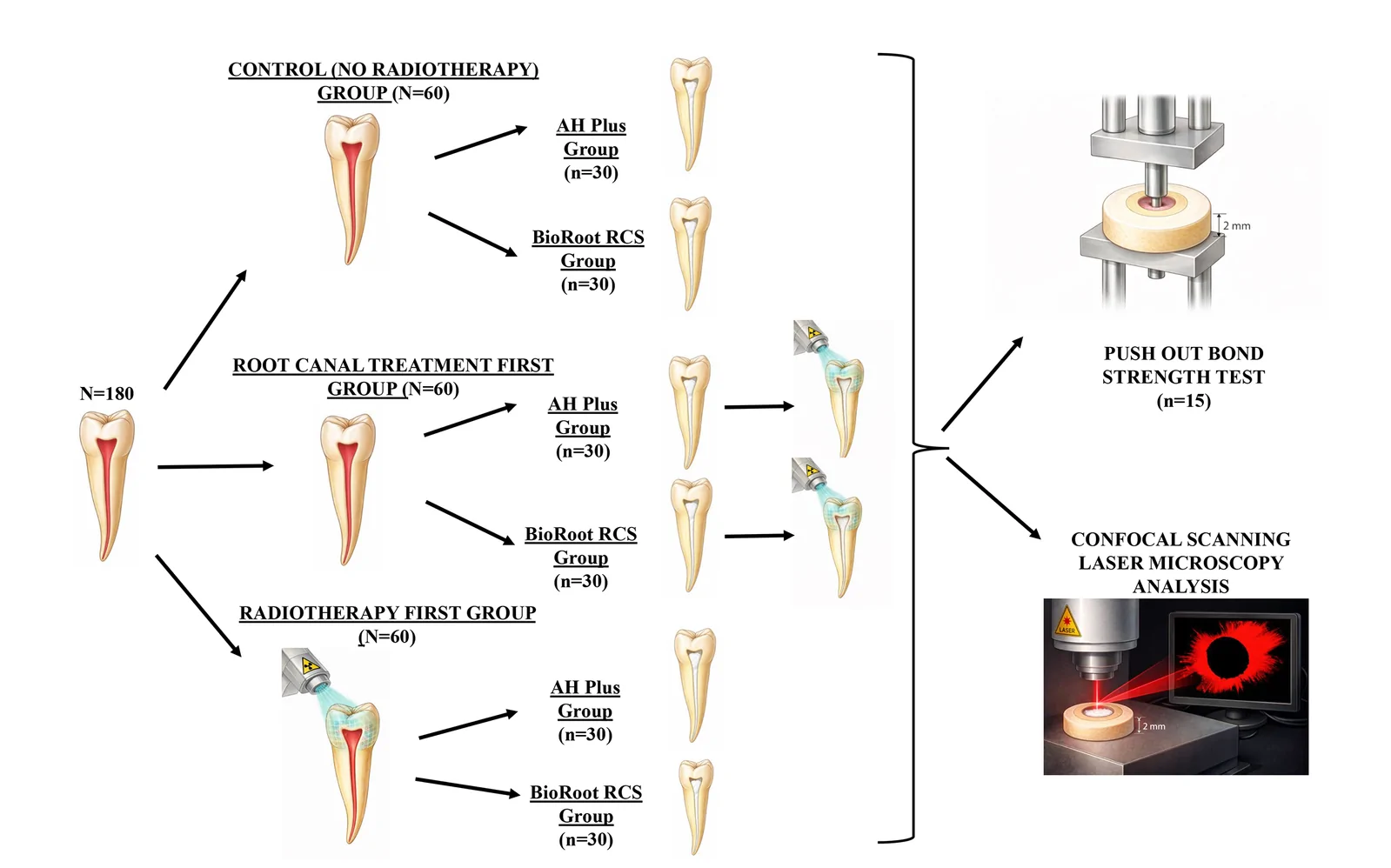
Experimental study design.

**Table 1 Table1:** Materials used in the study, including manufacturers, composition, lot numbers, and preparation/application procedures

Material	Manufacturer	Type	Main composition	LOT No.	Preparation/Application
AH Plus	Dentsply Sirona, York, PA, USA	Epoxy resin-based root canal sealer	Bisphenol-A epoxy resin, bisphenol-F epoxy resin, calcium tungstate, zirconium oxide, silica, amines	2301000302	Equal volumes (1:1) of Paste A and Paste B were mixed and applied into the canal by coating the master cone
BioRoot RCS	Septodont, Saint-Maur-des-Fossés, France	Calcium silicate-based bioceramic sealer	Tricalcium silicate, povidone, zirconium oxide, calcium chloride	B29306	One level spoonful of powder was mixed with five drops of liquid for ~60 s until a smooth homogeneous paste was obtained
Equia Forte HT	GC Corporation, Tokyo, Japan	Glass hybrid restorative material	Fluoroaluminosilicate glass, polyacrylic acid, water	220922A	Capsule was activated, mixed in a capsule mixer for 10 s, and applied according to the manufacturer’s instructions


### Root Canal Treatment and Obturation Procedures

Standard access cavities were prepared, and the canal patency was confirmed with a size 10 K-file (Dentsply-Maillefer, Switzerland) until the file tip was visible at the apical foramen. The working length was established by subtracting 1 mm from this point. The root canal shaping was performed using the VDW Rotate system (VDW, Munich, Germany), which enlarged the canals to a final size of 40/0.06 taper while maintaining the working length 1 mm short of the apex.

Throughout the instrumentation, the canals were irrigated with 2 ml of 5% sodium hypochlorite (NaOCl) (Wizard, Rehber Kimya, Turkey) after each file sequence using a 30-gauge side-vented needle positioned 2 mm short of the working length. The total volume of NaOCl used during canal preparation was approximately 12 ml per specimen. Following canal shaping, 2 ml of 17% ethylenediaminetetraacetic acid (EDTA) (Wizard, Rehber Kimya, Istanbul, Türkiye) was applied for 3 min to remove the smear layer, followed by 2 ml of 5% NaOCl for 1 min. A final flush with distilled water was performed, and the canals were dried using sterile paper points.

The root canal sealers were prepared in accordance with the manufacturers’ instructions (Table 1). In the groups with AH Plus, obturation was performed using a size 40 standardized gutta-percha master cone and accessory cones with the cold lateral compaction technique. In the groups with BioRoot RCS, obturation was performed using a size 40/.06 gutta-percha cone with the single-cone technique, according to the manufacturer’s recommendations. These protocols were selected to reflect the clinical procedures commonly used with each sealer. The coronal access was sealed with Equia Forte HT (GC, Tokyo, Japan). All specimens were incubated at 37°C with 100% humidity for 7 days for sealer setting,^31^ and subsequently, they were stored in distilled water at 37°C until the analyses were performed.

### Specimen Irradiation

Radiotherapy was carried out at Bezmialem Vakif University’s Oncology Clinic using a procedure designed to mimic the treatment regimen for HNC in adults. Throughout irradiation, the specimens were kept in distilled water to simulate intraoral conditions.^15^ A Cobalt-60 teletherapy device (Cirus, CIS Bio International, Gif-sur-Yvette, France) emitting 1.25 MeV gamma rays was used for the irradiation. The source-to-surface distance was maintained at 80 cm, and the field size was 20 × 30 cm. A cumulative dose of 60 Gy was delivered in doses of 2 Gy per day, 5 days per week, over a 6-week period and at a dose rate of 22.03 cGy/min. Quality assurance was verified via dosimetry, and a radiation oncologist, blinded to the dental protocols, oversaw the procedure.

### Confocal Laser Scanning Microscopy (CLSM) Analysis

The dentinal tubule penetration of the sealers was assessed at different root canal levels (n = 15 per subgroup). Firstly, the sealers were mixed with 0.1% rhodamine B dye (Sigma-Aldrich, St. Louis, MO, USA), and the prepared root canals were obturated. The specimens were positioned on an Isomet 1000 precision saw (Buehler, Lake Forest, IL, USA) to perform mesiodistal cuts perpendicular to the root axis and polished using a polishing device equipped with SiC abrasive papers with ascending grit levels (#600 to #2400) and under constant water cooling. Subsequently, the specimens were examined under a Leica TCS SP8 CLSM (Leica Microsystems, Mannheim, Germany) at 5× magnification using a wavelength of 514 nm. The digital images were analyzed with ImageJ software (NIH). The maximum penetration depth was determined based on the distance from the canal wall to the deepest point of sealer extension, following the protocol described by Bolles et al.^3^ All of the measurements were repeated twice to ensure accuracy.

The mean penetration depth was determined by averaging values from four standardized circumferential points (ie, at 12, 3, 6, and 9 o’clock), representing the buccal, mesial, lingual, and distal surfaces, respectively. A superimposed grid facilitated directional consistency across the specimens. All measurements were performed twice by the same blinded examiner, and the mean of the two measurements was used for the statistical analysis.

### Push-Out Bond Strength Test

The push-out bond strength was assessed at different root canal levels (n = 15 per subgroup). Firstly, each specimen was affixed to an acrylic resin plate using hot glue, ensuring that the root’s long axis remained parallel to the plate surface. The mounted specimens were positioned on an Isomet 1000 precision saw (Buehler, Lake Forest, IL, USA) under constant water cooling to perform mesiodistal cuts perpendicular to the root axis. From each root, three slices of approximately 2.0 ± 0.1 mm in thickness were obtained.

Each dentin slice was placed on a steel support with a central aperture and mounted onto a universal testing machine (AGS-X, Shimadzu, Tokyo, Japan). A compressive load was applied in the apico-coronal direction (crosshead speed: 1 mm/min) using stainless steel plungers (diameter: 1 mm and 0.6 mm) until bond failure occurred. The plunger diameters were selected according to the canal dimensions at each root level to ensure that the plunger contacted only the root-filling material without touching the surrounding dentin walls. Accordingly, the 1 mm plunger was used for the coronal and middle sections, whereas the 0.6 mm plunger was used for the apical sections. The dislodgement force (N) was recorded and converted into bond strength (MPa) using the following formula:

MPa = Force (N) / Bonded area (mm^2^) where the bonded surface area was calculated as follows:

Area = 2 × π × r × h,

with r representing the canal radius (mm) and h the slice thickness (mm).

Following the test, each dentin slice was examined using stereomicroscopy (20× magnification) to determine the mode of failure. The observations were classified into three categories: adhesive failure at the dentin–sealer interface, cohesive failure within the sealer or dentin, and mixed failure involving both types. These assessments were performed by a blinded examiner who was unaware of the group assignments.

### Statistical Analysis

SPSS software (v22.0, IBM, Chicago, IL, USA) was used for the statistical analyses. Data normality was determined via the Shapiro–Wilk test, and Levene’s test was used to confirm homogeneity of variances. As the data were normally distributed, intergroup comparisons were performed using a three-way ANOVA. The factors included in the model were radiotherapy timing (ie, Control, Before RT, and After RT), sealer type (AH Plus and BioRoot RCS), and root canal level (coronal, middle, and apical). The main effects and interaction effects were evaluated, and the effect sizes were expressed as eta squared (η^2^). Pairwise comparisons were performed using Bonferroni corrections. The distributions of the failure modes across the groups were compared using a chi-square test. Statistical significance was set at *p* < 0.05.

## Results

### Confocal Laser Scanning Microscopy (CLSM) Analysis

The maximum and mean dentinal tubule penetration depths, with standard deviations, are presented in Tables 2 and 3. Figure 2 presents confocal microscopy images of the sealer penetration at different root levels in all of the groups.

**Table 2 Table2:** Maximum penetration depths (μm) and standard deviations of the tested groups

	AH Plus					BioRoot RCS					p-value (AH Plus vs BioRoot RCS)			Effect size for (AH Plus vs BioRoot RCS)		
	Coronal	Middle	Apical	*p*	Effect size (η^2^)	Coronal	Middle	Apical	p	Effect Size (η2)	Coronal	Middle	Apical	Coronal	Middle	Apical
Control	284.676 ±102.54^7abA^	317.553 ±87.644^bA^	225.211 ±76.942^a^	0.018	0.037	272.187 ±117.905^AB^	288.09 ±112.306	215.527 ±84.934^A^	0.083	0.023	0.695	0.382	0.77	0.001	0.004	0
Before RT	208.107 ±50.091^AB^	246.999 ±73.268^A^	169.151 ±52.657	0.086	0.023	308.205 ±77.172^A^	280.441 ±100.528	272.222 ±90.324^A^	0.546	0.006	0.005	0.362	0.002	0.037	0.004	0.042
After RT	167.748 ± 95.405^B^	162.544 ±47.985^B^	166.105 ±99.032	0.987	0	214.079 ±93.409^aB^	224.934 ±73.075^a^	72.243 ±41.888^bB^	< 0.001	0.107	0.161	0.064	0.006	0.009	0.016	0.035
Effect size (η^2^)	0.061	0.088	0.019			0.035	0.019	0.152								
*p*	0.001	< 0.001	0.121			0.022	0.125	< 0.001								
*Different superscript lowercase letters indicate the significant differences within the same row.Different superscript uppercase letters indicate the significant differences within the same columns (*p* < 0.05). η^2^: effect size measure (eta squared).																

**Table 3 Table3:** Mean penetration depths (μm) and standard deviations of the tested groups

	AH Plus					BioRoot RCS					p-value (AH Plus vs BioRoot RCS)			Effect size for (AH Plus vs BioRoot RCS)		
	Coronal	Middle	Apical	*p*	Effect size (η^2^)	Coronal	Middle	Apical	p	Effect Size (η2)	Coronal	Middle	Apical	Coronal	Middle	Apical
Control	142.728 ±45.595^aA^	162.577 ±48.196^aA^	94.722 ±46.962^b^	0.002	0.056	149.917 ±59.28^aA^	185.21 ±89.48^a^	99.006 ±51.6^bA^	< 0.001	0.078	0.708	0.266	0.831	0.001	0.006	0
Before RT	139.211 ±48.831^abA^	159.143 ±54.709^aA^	89.406 ±29.675^b^	0.004	0.051	201.075 ±69.103^aB^	168.719 ±60.495^ab^	128.643 ±45.806^bA^	0.002	0.057	0.004	0.665	0.055	0.037	0.001	0.017
After RT	58.352 ±26.782^B^	73.574 ±22.131^B^	79.27 ±40.98	0.551	0.006	143.527 ±74.788^aB^	136.874 ±40.924^a^	37.263 ±17.444^bB^	< 0.001	0.139	< 0.001	0.002	0.039	0.079	0.044	0.02
Effect size (η^2^)	0.099	0.101	0.003			0.042	0.027	0.092								
*p*	< 0.001	< 0.001	0.728			0.011	0.051	< 0.001								
*Different superscript lowercase letters indicate the significant differences within the same row. Different superscript uppercase letters indicate the significant differences within the same columns (*p* < 0.05). η^2^: effect size measure (eta squared).																

**Fig 2 Fig2:**
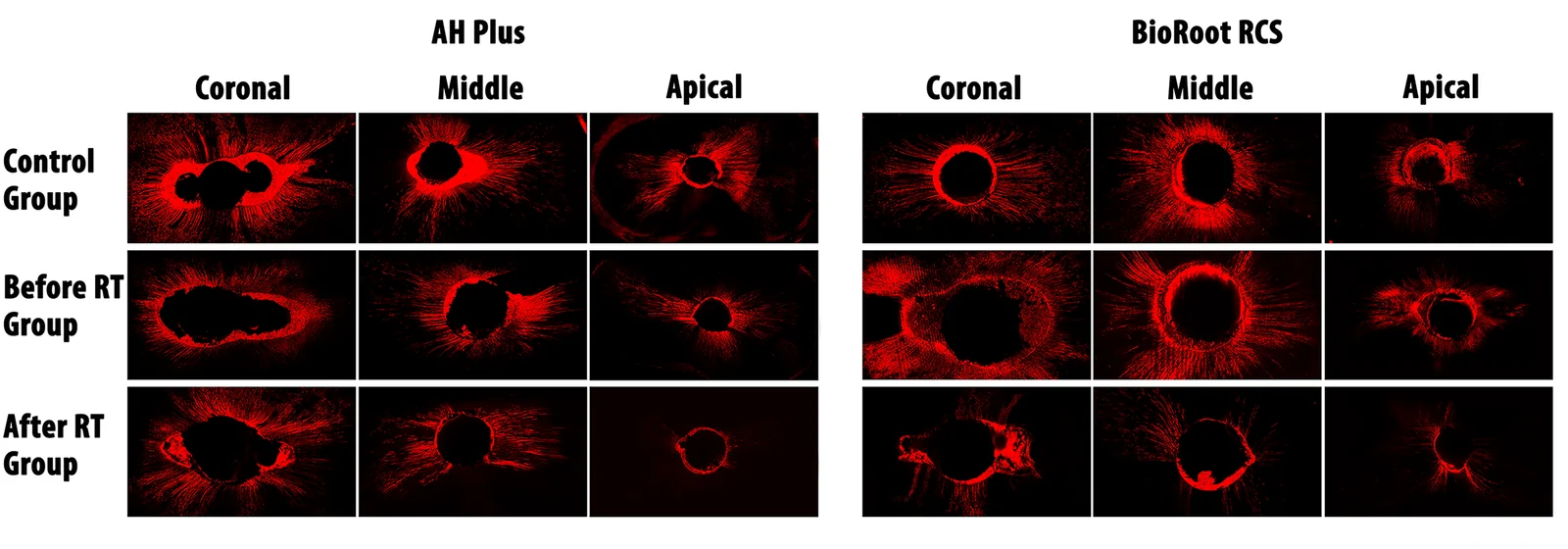
Confocal microscopy images of sealer penetration at different root levels in all groups.

For AH Plus, in the coronal thirds, the After RT group showed significantly lower penetration depths compared to the Control group. Moreover, in the middle thirds, the After RT group showed significantly lower penetration depths than the Control and Before RT groups (*p* < 0.05). However, in the apical thirds, the penetration depths were found to be similar across all groups (*p* > 0.05).

For BioRoot RCS, in the coronal thirds, the After RT group showed significantly lower penetration depths compared to the Control group (*p* < 0.05). In the middle thirds, no significant differences were detected among the groups (*p* > 0.05). Finally, in the apical thirds, the After RT group showed significantly lower penetration depths than the Control and Before RT groups (*p* < 0.05).

In terms of the root canal levels, the penetration depth in the apical thirds was found to be significantly lower than in the coronal and middle thirds in the After RT group with BioRoot RCS (*p* < 0.05).

In terms of the root canal sealers, for the coronal thirds in the Before RT groups, BioRoot RCS showed significantly greater penetration depths than AH Plus. Conversely, for the After RT groups, BioRoot RCS showed significantly lower penetration depths than AH Plus (*p* < 0.05).

### Push-Out Bond Strength

The push-out bond strength values with standard deviations are summarized in Table 4.

**Table 4 Table4:** Push-out bond strength values (MPa) and standard deviations of the tested groups

	AH Plus					BioRoot RCS					p-value (AH Plus vs BioRoot RCS)			Effect size for (AH Plus vs BioRoot RCS)		
	Coronal	Middle	Apical	*p*	Effect size (η^2^)	Coronal	Middle	Apical	p	Effect Size (η2)	Coronal	Middle	Apical	Coronal	Middle	Apical
Control	2.239 ±0.795^aA^	1.717 ±0.791^a^	6.217 ±3.22^bA^	< 0.001	0.233	5.145 ±1.846^aA^	2.693 ±1.261^bA^	4.784 ±2.323^aA^	< 0.001	0.085	< 0.001	0.085	0.013	0.097	0.012	0.026
Before RT	3.138 ±1.858^A^	2.025 ±0.453	2.778 ±1.038^B^	0.135	0.017	1.971 ±1.418^B^	2.391 ±0.956^A^	3.245 ±2.138^B^	0.115	0.018	0.048	0.533	0.436	0.016	0.002	0.003
After RT	0.697 ±0.502^aB^	0.995 ±0.892^ab^	2.294 ±1.611^bB^	0.012	0.037	1.027 ±0.755^B^	0.91 ±1.033^B^	1.679 ±2.15^C^	0.428	0.007	0.566	0.883	0.318	0.001	0	0.004
Effect size (η^2^)	0.075	0.015	0.188			0.193	0.044	0.098								
*p*	< 0.001	0.176	< 0.001			< 0.001	0.005	< 0.001								
*Different superscript lowercase letters indicate the significant differences within the same row.Different superscript uppercase letters indicate the significant differences within the same columns (*p* < 0.05). η^2^: effect size measure (eta squared).																

For AH Plus, in the coronal thirds, the bond strength was significantly greater in the Control and Before RT groups compared to the After RT group (*p* < 0.05). In the apical thirds, the Control group demonstrated significantly higher bond strength than the Before RT and After RT groups (*p* < 0.05). However, in the middle thirds, no significant differences in bond strength were found among the groups (*p* > 0.05).

For BioRoot RCS, in the coronal and apical thirds, the bond strength was significantly greater in the Control group than in the Before RT and After RT groups (*p* < 0.05). Additionally, in the middle thirds, the Control and Before RT groups showed significantly higher bond strength than the After RT group (*p* < 0.05). Finally, in the apical thirds, the Before RT group exhibited significantly higher bond strength than the After RT group (*p* < 0.05).

In terms of the root canal levels, the bond strength in the apical thirds was found to be significantly higher than in the coronal and middle thirds in the After RT groups with AH Plus (*p* < 0.05).

In terms of the root canal sealers, for the coronal thirds in the Before RT groups, AH Plus exhibited significantly greater bond strength than BioRoot RCS (*p* < 0.05).

The three-way ANOVA model also revealed significant interaction effects among the study variables. For the maximum penetration depth, significant interactions were observed between radiotherapy timing and sealer type (*p* = 0.002) and among radiotherapy timing, sealer type and root canal level (*p* = 0.012). For the mean penetration depth, significant interactions were found between sealer type and root canal level (*p* = 0.009) and among radiotherapy timing, sealer type and root canal level (*p* = 0.003). For the push-out bond strength, significant interactions were detected between radiotherapy timing and root canal level (*p* < 0.001), between sealer type and root canal level (*p* = 0.031), and among radiotherapy timing, sealer type and root canal level (*p* < 0.001).

### Failure Mode Analysis

The distributions of the failure modes are summarized in Table 5. For both sealers, in the Control and Before RT groups, mixed-type failures were the most common, followed by cohesive failures. In contrast, adhesive failures occurred more frequently in the After RT groups. The chi-square analysis revealed a significant difference in failure mode distribution only in the Control group with BioRoot RCS (*p* = 0.002), whereas no significant differences were observed in the other groups (*p* > 0.05).

**Table 5 Table5:** Distribution of failure modes (%) and statistical comparisons among root canal levels after the push-out bond strength test

Groups	Root canal levels	Adhesive (%)	Cohesive (%)	Mixed (%)	*p* value
**Control – AH Plus**	Coronal	0.0	46.7	**53.3**	0.294
	Middle	0.0	20.0	**80.0**	
	Apical	0.0	33.3	**66.7**	
**Before RT – AH Plus**	Coronal	0.0	6.7	**93.3**	0.549
	Middle	0.0	13.3	**86.7**	
	Apical	0.0	20.0	**80.0**	
**After RT – AH Plus**	Coronal	20.0	0.0	**80.0**	0.565
	Middle	20.0	6.7	**73.3**	
	Apical	20.0	13.3	**66.7**	
**Control – BioRoot RCS**	Coronal	13.3	40.0	**46.7**	**0.002**
	Middle	0.0	33.3	**66.7**	
	Apical	0.0	0.0	**100.0**	
**Before RT – BioRoot RCS**	Coronal	13.3	**46.7**	40.0	0.275
	Middle	13.3	13.3	**73.3**	
	Apical	20.0	20.0	**60.0**	
**After RT – BioRoot RCS**	Coronal	13.3	6.7	**80.0**	0.065
	Middle	26.7	6.7	**66.7**	
	Apical	**60.0**	0.0	40.0	


## Discussion

This *in vitro* study evaluated the influence of radiotherapy timing on the dentinal tubule penetration and push-out bond strength of an epoxy-resin-based sealer and a bioceramic sealer.

The null hypothesis was rejected, as radiotherapy timing significantly influenced sealer-dentin adhesion performance, and the outcomes varied by root canal level and material type. Notably, radiotherapy administered after root canal obturation had a more detrimental effect on both tubule penetration and bond strength.

Radiotherapy for HNC is typically delivered in fractionated doses to maximize tumor control while allowing normal tissues to recover from sublethal damage.^5^ The posterior teeth, particularly the mandibular premolars, have been reported to receive relatively high doses of radiation during radiotherapy, depending on the tumor location and treatment plan.^5,22,28
^ Accordingly, a cumulative dose of 60 Gy was selected in the present study to simulate clinically relevant radiotherapy conditions, as informed by previous experimental data.^21^


The dentinal tubule penetration of sealers is influenced by various factors, including the dentin morphology, smear layer removal, and the physicochemical properties of the sealer itself, such as its flowability, solubility, and setting characteristics.^3,9
^ Sealer penetration is commonly evaluated in laboratory settings using CLSM or scanning electron microscopy (SEM). CLSM offers the advantage of enabling the non-destructive, 3D visualization of fluorescently labeled materials at varying tissue depths. Rhodamine B at a 0.1% concentration is widely accepted for fluorescent tagging, as it ensures adequate imaging resolution without altering the sealer’s handling or setting properties.^25^ In the present study, both the maximum and mean penetration depths were assessed to provide a comprehensive evaluation of tubular infiltration.^3,9,24,25
^ However, it should be noted that isolated deep penetration values may lack clinical relevance if the sealer is not evenly distributed circumferentially throughout the canal.^3^


In terms of radiotherapy timing, the CLSM analysis in our study showed that AH Plus and BioRoot RCS had significantly higher dentinal tubule penetration in the Control group compared to the After RT group in the coronal thirds. The reduced penetration observed when radiotherapy was administered after root canal obturation may be attributed to radiation-induced alterations causing the potential degradation of the sealer-dentin interfaces. In addition to the direct structural and chemical alterations in dentin induced by radiation, such as collagen degradation and changes in the organic matrix, radiation-induced dehydration may also play a critical role in the observed outcomes. Indeed, for calcium silicate–based sealers, which rely on moisture-dependent hydration reactions for setting and biomineralization, alterations in the moisture conditions of the dentin may influence their setting behavior and interfacial adaptation.^7,21
^ Therefore, the inferior performance observed in the After RT groups may not only be attributed to dentin-related changes but also to the potential effect of radiation-induced dehydration on the physicochemical behavior of the sealer. The alterations in dentinal tubules caused by irradiation may affect the integrity of the previously formed sealer tags and reduce the measurable penetration depth.

Notably, for BioRoot RCS, the reduction in tubule penetration for the After RT group compared to the Control group was also observed in the apical third. Furthermore, when the root canal levels were compared within the After RT group, the penetration depth in the apical third was significantly lower than in the coronal and middle thirds. The apical third has fewer and narrower tubules, increased dentin sclerosis, and reduced irrigant efficacy, which may limit smear layer removal and sealer penetration,^4,6,19
^ thereby contributing to the lower penetration values. Calcium silicate-based sealers rely on moisture-dependent reactions and ionic exchange with dentin for effective interaction.^1,6,18
^ As such, radiation-induced dehydration and alterations in the dentin may disproportionately compromise their penetration at this level.^14,18
^


Some previous studies have reported that radiation is associated with a decrease in the bond strength of root canal sealers.^17,26
^ In our study, in terms of radiotherapy timing, both sealers exhibited significantly reduced bond strength when radiotherapy was administered after obturation, particularly in the coronal and apical thirds. These findings were further supported by the stereomicroscopic analysis, which revealed a higher incidence of adhesive failures in the After RT group. Radiation-induced alterations in dentin, including collagen degradation, free radical formation, and changes in the organic matrix, may compromise the sealer-dentin interface. In turn, these alterations may contribute to less favorable bonding conditions and may be responsible for the lower push-out bond strength values observed in irradiated specimens. These findings are in line with a previous study reporting compromised adhesion, especially when radiotherapy is applied after endodontic treatment.^8^ However, in contrast, Yaduka et al^38^ evaluated the influence of radiotherapy on the push-out bond strength of sealers and reported that radiotherapy prior to endodontic treatment decreased the bond strength of AH Plus but not BioRoot RCS. These differences in findings could be attributed to the differences in the obturation techniques and methodologies used across the studies.

It should be emphasized that the dentinal tubule penetration and push-out bond strength, although potentially related, do not represent the same biomechanical phenomenon. Dentinal tubule penetration primarily reflects the ability of a sealer to infiltrate the dentinal substrate, whereas push-out bond strength is a mechanical parameter influenced by multiple factors, including interfacial adaptation, material properties, dentin condition, and physicochemical interactions at the sealer-dentin interface.^27^ Consequently, differences in the penetration depth should not be interpreted as direct evidence of corresponding differences in the bond strength.

In our study, in the apical thirds, the bond strength and tubule penetration were higher when radiotherapy was administered prior to rather than after obturation for BioRoot RCS, although no significant differences were observed between radiotherapy timings for AH Plus. Calcium silicate-based sealers interact with dentin through biomineralization processes and form a mineral infiltration zone, and this process depends on the chemical interaction between the sealer and dentin.^1,29,30
^ Radiotherapy after obturation may adversely affect the integrity of this interfacial layer and thereby influence the interaction between the sealer and dentin. In contrast, epoxy-resin-based sealers such as AH Plus primarily rely on micromechanical retention and covalent bonding,^10^ which may be less affected by the timing of radiotherapy.

In the Before RT groups, AH Plus demonstrated significantly higher bond strength than BioRoot RCS in the coronal thirds despite exhibiting lower dentinal tubule penetration. This difference may be attributed to the distinct adhesion mechanisms of the two sealers. Specifically, AH Plus is known to bond with dentinal collagen through chemical interactions between the epoxy resin groups and exposed amine groups, contributing to its higher bond strength.^10^ In contrast, radiotherapy may impair the interfacial interactions through biomineralization that are required for BioRoot RCS. Although calcium silicate-based sealers may exhibit deeper dentinal tubule penetration due to their flow characteristics and smaller particle size, the dentinal tubule penetration and push-out bond strength represent different aspects of the sealer-dentin interaction.^10,12
^ Indeed, dentinal tubule penetration is primarily a morphologic and interfacial parameter, whereas push-out bond strength is a mechanical outcome influenced by multiple factors.^29,36
^ Therefore, greater penetration should not be interpreted as direct evidence of superior bonding performance. It should be noted that AH Plus and BioRoot RCS were used according to their respective manufacturers’ recommendations and commonly adopted clinical protocols. Therefore, any direct comparison between the two materials should be interpreted with caution, as the observed differences may reflect not only material-related characteristics but also the influence of the obturation technique.

This study has some limitations that should be acknowledged. Firstly, the use of single-rooted teeth may limit the extrapolation of the findings to teeth with more complex root canal anatomies. In addition, variations in tooth age and reasons for extraction may have influenced the dentin structure and the behavior of the sealers.^29^ As this work was an *in vitro* study, the experimental conditions cannot fully replicate the biological and mechanical environment of the oral cavity. Another limitation of the study is that multiple slices obtained from the same tooth were treated as independent observations, which may not fully account for potential clustering effects. Furthermore, different obturation techniques were used for the tested sealers in accordance with the manufacturers’ recommendations and commonly adopted clinical protocols. Although this approach was intended to reflect the clinical use of each material, the obturation technique may influence the sealer distribution, sealer thickness, adaptation to the canal walls, dentinal tubule penetration, and push-out bond strength. Therefore, the direct comparisons between AH Plus and BioRoot RCS should be interpreted with caution, as the observed differences may be related not only to the intrinsic properties of the sealers but also to the obturation protocol employed. Moreover, the dentinal tubule penetration was evaluated using CLSM from a single perspective and based on four standardized circumferential measurement points. Although this methodology allowed for standardized comparisons among the specimens, it may not fully capture the heterogeneity of sealer penetration throughout the entire canal circumference. In addition, a formal intra-examiner reliability analysis was not performed for all specimens, and this should be considered when interpreting the CLSM findings. Additional quantitative analyses, such as percentage-based evaluations, could provide more comprehensive information. Therefore, further in vivo and long-term studies are needed to confirm the clinical relevance of these findings.

## Conclusion

Within the limitations of this study, it can be concluded that radiotherapy timing significantly influenced sealer-dentin adhesion, and this effect varied by root canal levels and material types. Radiotherapy applied after obturation adversely affected the sealer-dentin interaction, resulting in reduced tubule penetration and bond strength and a higher incidence of adhesive failures, particularly for BioRoot RCS.

### Acknowledgments

The authors deny any conflicts of interest related to this study. This study was presented as an oral presentation at the 22nd ESE Biennial Congress, held from September 3–6, 2025, in Paris, France. It was supported by the Bezmialem Vakıf University Scientific Research Projects Coordination Unit Research Fund (Project No: 20221209). The authors thank Assistant Professor Sevilay Karahan for her help with statistical analysis.

#### Clinical significance

The timing of radiotherapy may influence the adhesion performance of root canal sealers. The findings of this study suggest that completing endodontic treatment before radiotherapy, when clinically feasible, may help preserve the sealer penetration and bond strength.

#### Ethical statement

The study received approval from the Scientific Research Ethics Committee of Bezmialem Vakif University (E-54022451-050.05.04-86178).

## References
